# Humanity on the Battlefield

**Published:** 1907-06-22

**Authors:** 


### Humanity on the Battlefield.

In 1902 the Empress Marie Feodorovna of Russia, whose interest in ambulance work has been manifested on many occasions in an eminently practical fashion, presented to the International Red Cross Conference the sum of 100,000 roubles; the interest on which sum she desired should be devoted to a prize for the development of Red Cross work. The rules of the Marie Feodorovna prizes have been drawn up by a committee of the International Red Cross Conference, and under these the prizes are awarded quinquennially for developments along lines specifically suggested by the jury. This year the prizes will be given "for inventions relating to the discovery and the raising of the wounded on the battlefield, on land, and at sea: the methods of transporting the wounded to the nearest posts for medical aid as rapidly as possible and with the smallest amount of suffering to them.

How keen has been the competition among the various Red Cross Associations to secure the munificent awards that the generosity of the Russian Empress has made it possible for the Conference to grant, anyone visiting last week's Exhibition at Earl's Court would have been able to see for himself. The ingenuity displayed in some of the models exhibited is only surpassed, perhaps, by the skill shown in perfecting the death-dealing apparatus whose employment in modern warfare makes the work of the ambulance so onerous, and in some respects so dangerous. The exhibition showed how greatly we have progressed in Red Cross work since the times of Larrey, and we believe that future exhibitions would gain in practical, as they would undoubtedly gain in educative value, if the committee took means to enable the visitor and the student to compare modern Red Cross appliances with those formerly in vogue. A few years seem to make as much difference in these things as they do in the fittings of a war ship: there is a fashion in ambulances as there is in quick-firing guns. This is well seen in the excellent model of the Russian Red Cross train (No. 175) which presents several points of advance upon the elaborate hospital trains used by us in the South African war, showing a better economy of space and a greater adaptation of the latest improvements to the necessities of corridor carriages. An equally interesting exhibit was that shown by the same society, the Yourte Tent, which is used by the Siberian Nomads, and combines efficiency with extreme lightness and simplicity. The key-note of the various models, indeed, appears to have been simplicity; no unnecessary screw, bolt, rest, or support is to be seen, and strength and firmness are in no case sacrificed to appearance or fad. Foreign working models predominated, but it was satisfactory to note that the English exhibits, though numerically weaker than those of foreign competitors, are thoroughly worthy of comparison with the latter, and that the larger number of British exhibitors entered for the competition for the Marie Feodorovna prizes. The exhibits of the Grand Priory of St. John, of Colonel Hathaway, and of Mr. Harman Luce proved how actively our own Red Cross associations are attempting to deal with these difficult problems of the transport and care of the wounded on the battlefield.

In all these exhibits the lessons learned during the last two great wars between civilised nations have been steadily kept in mind by the inventors. The excessive jolting which was so marked a feature in old military ambulances has been overcome by the use of ingenious swivel supports: the requirements of modern aseptic surgery have been met by providing operating carriages fitted like the most up-to-date theatres, with the corners and projections rounded off, and every facility for the free ventilation of the carriage or wagon fully guaranteed. As a record of Red Cross work and ingenuity the exhibition is a revelation to most of us. As a sign of how gradually we are approaching to a solution of a problem that is as important as it is difficult it is both encouraging and satisfactory—encouraging since it proves that Red Cross organisation all the world over is active in facing the problem, and satisfactory because it convinces the observer that some of the exhibitors have come very close to finding a solution of many of the difficulties.

